# New Dandelion Algorithm Optimizes Extreme Learning Machine for Biomedical Classification Problems

**DOI:** 10.1155/2017/4523754

**Published:** 2017-09-11

**Authors:** Xiguang Li, Shoufei Han, Liang Zhao, Changqing Gong, Xiaojing Liu

**Affiliations:** School of Computer, Shenyang Aerospace University, Shenyang 110136, China

## Abstract

Inspired by the behavior of dandelion sowing, a new novel swarm intelligence algorithm, namely, dandelion algorithm (DA), is proposed for global optimization of complex functions in this paper. In DA, the dandelion population will be divided into two subpopulations, and different subpopulations will undergo different sowing behaviors. Moreover, another sowing method is designed to jump out of local optimum. In order to demonstrate the validation of DA, we compare the proposed algorithm with other existing algorithms, including bat algorithm, particle swarm optimization, and enhanced fireworks algorithm. Simulations show that the proposed algorithm seems much superior to other algorithms. At the same time, the proposed algorithm can be applied to optimize extreme learning machine (ELM) for biomedical classification problems, and the effect is considerable. At last, we use different fusion methods to form different fusion classifiers, and the fusion classifiers can achieve higher accuracy and better stability to some extent.

## 1. Introduction

Nature has evolved over hundreds of millions of years, showing the perfect efficiency and magic. People learn a lot from the study of natural systems and use them to develop new algorithms and models to solve complex problems. Therefore, imitation of biological intelligence behavior, drawing on its intelligent mechanism, making many new ways to solve complex problems continue to emerge. Through the modeling of natural intelligence, a number of intelligent algorithms have been proposed, including genetic algorithms [1], ant colony algorithm [2], particle swarm algorithm [3, 4], center gravity search algorithm [5, 6], and quantum computing [7]. Each intelligent algorithm corresponds to an actual source of inspiration. For example, DNA calculations are based on a double helix structure proposed by Watson and Crick who win the Nobel Prize in physiology or medicine and a polymerase linker response proposed by a Nobel Prize winner Mullis [8]. Artificial bee colony algorithm is based on the decoding of the bees dance behavior [9]. Artificial immune algorithm is based on immune network theory [10]. The bat algorithm is presented by simulating the bat echo positioning behavior [11]. Inspired by observing fireworks explosion, enhanced fireworks algorithm is proposed for global optimization of complex functions [12]. In recent years, many intelligent algorithms have been applied in engineering problems successfully [13–20], which not only reduce the time consumed but also can guarantee better performance than manual adjustment.

The above-mentioned intelligent algorithms are all parallel to search for the optimal solution. However, the individuals in them are using the same mechanism in the process of searching. In this paper, inspired by the behavior of dandelion sowing, a novel swarm intelligence algorithm called dandelion algorithm (DA) is proposed for function optimization. Such an optimization algorithm has advantages such as a simple computational process and ease of understanding. In DA, dandelion populations are divided into two subpopulations, suitable for sowing and unsuitable for sowing, and then perform different sowing ways for different subpopulations. Meanwhile, another way of sowing is to carry out the subpopulation which is suitable for sowing, in order to avoid falling into the local optimum. To validate the performance of the proposed DA, in our simulation, we apply the twelve standard functions and compare the proposed algorithm (DA) with bat algorithm (BA), particle swarm optimization (PSO), and enhanced fireworks algorithm (EFWA). The results show that the proposed algorithm has better overall performance on the test functions.

Extreme learning machine is an advanced neural network [21]. The input weight and the hidden layer bias are randomly generated according to the number of input neurons and hidden layer nodes, and the output weight matrix is calculated according to the Moore-Penrose generalized inverse of the hidden layer output matrix. Although the extreme learning machine has many advantages over traditional neural networks, it causes its instability due to its random input weight and hidden layer bias. In order to obtain higher accuracy, this paper proposes a method to optimize the extreme learning machine with proposed algorithm (DA) for biomedical classification problems. Moreover, we combine multiple classifiers to form a fusion classifier with different fusion methods for biomedical classification, and the results show that it has better performance.

The paper is organized as follows. In Section 2, the dandelion algorithm is introduced. The simulation experiments and results analysis are given in detail in Section 3. Using DA to optimize ELM and combining multiple classifiers to form a fusion classifier with different fusion methods are presented in Section 4. Finally, the conclusion is summarized in final part.

## 2. Dandelion Algorithm

### 2.1. DA Framework

In DA, we assume that the earth is divided into two types: suitable for dandelion sowing and unsuitable for dandelion sowing, and the dandelion living in suitable environment is called core dandelion (CD); on the contrary, the dandelions except for the core dandelion are called assistant dandelions (AD).

Without loss of generality, consider the following minimization problem:

(1) $$\begin{array}{c} y=\min f\left(\;x\;\right); \end{array}$$

the objective is to find an optimal *x* with minimal evaluation (fitness) value.

**Figure 1:**
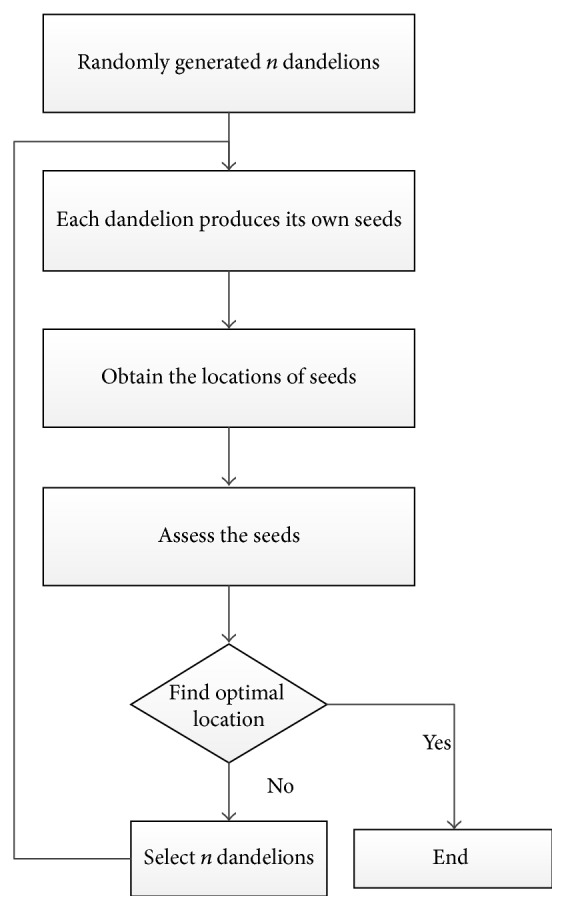
Framework of dandelion algorithm.

When a dandelion is sown, the seeds of dandelion will be scattered around the dandelion. In our view, the process of dandelion sowing can be seen to search an optimal in a particular space around a point. For example, now we need to find a point *x* to satisfy *y* = *f*(*x*); then using the dandelion to sow the seeds in potential space until finding a point is infinitely close to the point *x*. Mimicking the process of dandelion sowing, a rough framework of the DA is depicted in Figure 1.

In DA, with each generation of sowing, firstly, we need to select *n* dandelions; that is to say, here we have *n* dandelions to sow. Then after sowing, the locations of seeds are obtained and assessed. The algorithm will stop when the optimal location is found. Otherwise, the algorithm needs to select other *n* dandelions from the all seeds and dandelions for the next generation of sowing.

From Figure 1, we can see that the process of sowing and selection strategy are important for DA, and they are, respectively, described in detail in the following.

### 2.2. Design of DA

In this section, we will introduce the design of the various operators of the dandelion algorithm and the mathematical model in detail. In the DA, we assume that there are only two types of dandelion: core dandelion (CD) and assistant dandelions (AD), and different types of dandelions perform different sowing ways. Meanwhile, another way of sowing, called mutation sowing, is designed to avoid falling into local optimum. Finally, the selection strategy is designed to select dandelions to enter the next generation.

To sum up, the dandelion algorithm consists of normal sowing, mutation sowing, and selection strategy.

#### 2.2.1. Normal Sowing

In the DA, we stipulate that the core dandelion can produce more seeds, and the assistant dandelion produces less seeds, because the land with the core dandelion is suitable for the seeds to grow. The number of seeds produced by the sowing is calculated based on its relative dandelions fitness values in the dandelion population. Assume that the maximum number of seeds is* max* and the minimum number of seeds is* min*; the number of seeds *M*_*i*_ for each dandelion *X*_*i*_ is calculated as follows.

(2) $$\begin{array}{c} M_{i}=\left\{\begin{array}{cc} max\times \frac{f_{max}-f\left(x_{i}\right)+\varepsilon }{f_{max}-f_{min}+\varepsilon } & M_{i}>min\\ min & M_{i}\leq min, \end{array}\right. \end{array}$$

where *f*_max_ = max⁡(*f*(*x*_*i*_)), *f*_min_ = min⁡(*f*(*x*_*i*_)), and *ε* is the machine epsilon to avoid the denominator which is equal to 0.

From (2), for the minimization problem, we can see that the dandelion with small fitness value will sow more seeds, and the dandelion with large fitness value will sow less seeds but can not be less than the minimum number of seeds.

In DA, dandelions are divided into two types: assistant dandelions and core dandelion; the core dandelion (CD) is the dandelion with the best fitness, and it is calculated by

(3) $$\begin{array}{c} X_{CD}=\min f\left(\;x_{i}\;\right). \end{array}$$

The calculation of the radius of the assistant dandelions and the core dandelion is different. The assistant dandelions' sowing radius (except for CD) is calculated by

(4) Rit=UB−LBt=1w×Rit−1+XCD∞−xi∞otherwise,

where* t* is the the number of iterations, UB is upper bound of the function, LB is lower bound of the function, and infinite norm is the maximum of all dimensions.

From (4), at the beginning of the algorithm, the sowing radius for the assistant dandelions is set to the diameter of the search space. After that, it is set to difference of distance between current assistant dandelion and core dandelion; here we use infinite norm to measure distance. Moreover, in order to slow down the convergence rate to improve the global search performance, on the basis of the above, we added the sowing radius of assistant dandelion in the previous generation, and the *w* is a weight factor, to adjust the impact of the sowing radius of previous generation on the current sowing radius dynamically. The weight factor *w* is designed as follows.

(5) $$\begin{array}{c} w=1-\frac{Fe}{{Fe}_{max}}, \end{array}$$

where Fe is the current function evaluations and Fe_max_ is the maximum number of function evaluations. It can be seen that the value of *w* changed from large to small, it means that the sowing radius of the previous generation has less and less impact on the current sowing radius.

But for the CD, it is another way to calculate the sowing radius, which is adjusted based on the CD in the last generation; it is designed as follows.

(6) $$\begin{array}{c} R_{CD}\left(\;t\;\right)=\left\{\begin{array}{cc} UB-LB & t=1\\ R_{CD}\left(t-1\right)\times r & a=1\\ R_{CD}\left(t-1\right)\times e & a\neq 1, \end{array}\right. \end{array}$$

where *R*_CD_(*t*) is the sowing radius of the CD in generation *t*. At the beginning of the algorithm, the sowing radius for the CD is also set to the diameter of the search space. *r* and *e* are the withering factor and growth factor, respectively, and *a* reflects the growth trend, which is calculated by

(7) $$\begin{array}{c} a=\frac{f_{CD}\left(t\right)+\varepsilon }{f_{CD}\left(t-1\right)+\varepsilon }, \end{array}$$

where *ε* is the machine epsilon to avoid the denominator which is equal to 0. When *a* = 1, it means that the algorithm does not find a better solution, and the place is not suitable for sowing; thus, we need to reduce the sowing radius, and the withering factor *r* is designed to describe this situation; of course *r* can not be too small; the value should be in [0.9, 1). On the contrary, when *a* ≠ 1, it means that the algorithm finds a better solution than last generation, and the place is suitable for sowing, and the sowing radius should be enlarged, which can speed up the convergence rate; based on this, the growth factor *e* is proposed; of course *e* can not be too large; the value should be in (1, 1.1].

Algorithm 1 describes the process of the normal sowing in DA. *X*_min_^*k*^ and *X*_max_^*k*^ refer to the lower and upper bounds of the search space in dimension *k*.

#### 2.2.2. Mutation Sowing for the Core Dandelion

In order to avoid falling into the local optimal and keep the diversity of the population, another way to sow, called mutation sowing, is proposed for the CD. It is defined as

(8) $$\begin{array}{c} X_{CD}^{\prime }=X_{CD}\times \left(\;1+Levy\left(\;\;\;\right)\;\right), \end{array}$$

where Levy( ) is a random number generated by the Levy distribution, and it can be calculated with parameter *β* = 1.5.

Algorithm 2 is performed for mutation sowing for CD to generate location of seeds. This algorithm is performed *N*_*m*_ times (*N*_*m*_ is a constant to control the number of mutation seeds).

#### 2.2.3. Selection Strategy

In the DA, it requires that the current best location is always kept for the next iteration. In order to keep the diversity, the remaining locations are selected based on disruptive selection operator. For location *X*_*i*_, the selection probability *p*_*i*_ is calculated as follows.

(9) pi=fi∑n=1SNfnfi=fi−favg,

where *f*_*i*_ is the fitness value of the objective function, *f*_avg_ is the mean of all fitness values of the population in generation *t*, and SN is the set of all dandelions (dandelions, normal seeds, and mutation seeds).

The selection probabilities determined by this method can give both good and poor individuals more chances to be selected for the next iteration, while individuals with mid-range fitness values will be eliminated. This method can not only keep the diversity of the population but also reflect the better global searching ability.

### 2.3. Summary

Assume that the number of dandelion populations is *N*.  Algorithm 3 summarizes the framework of the DA. During each sowing, two types of seeds are generated, respectively, according to Algorithms 1 and 2. Firstly, the number of seeds and sowing radius are calculated based on the quality of the corresponding dandelion. Moreover, another type is designed with a Levy mutation, which can help to avoid falling into local optimum. After that, *N* locations are selected for the next generation. In the DA, we assume that the total number of normal seeds is *N*_*s*_, and the number of mutation seeds is *N*_*m*_. So approximate *N* + *N*_*s*_ + *N*_*m*_ function evaluations are done in each generation. Suppose the optimum of a function can be found in *t* generations; then we can deduce that the complexity of the DA is *O*(*t* × (*N* + *N*_*s*_ + *N*_*m*_)).

## 3. Experiments

To assess the performance of DA, it is compared with BA, EFWA, and PSO.

### 3.1. Experiment Settings

The parameters of DA, BA, EFWA, and PSO are setting as Table 1, and the settings are applied in all the comparison experiments.

In Table 1, *n* is population size, *A* is the loudness, *r* is the the rate of pulse emission, *m* is the total number of sparks, and *a* and *b* are fixed constant parameters that confine the range of the population size. *A*_max_ is the the maximum explosion amplitude and *n*_*m*_ is the number of mutation dandelions.

In the experiment, the function of each algorithm is repeated 51 times, and the final results after the 300,000 function evaluations are presented. In order to verify the performance of the algorithm proposed in this paper, we use the 12 different types of test functions, which are listed in Table 2 and their expressions are listed in the appendix.

Finally, we use Matlab R2014a software on a PC with a 3.2 GHz CPU (Intel Core i5-3470), and 4 GB RAM, and Windows 7 (64 bit).

### 3.2. Comparison Experiments among the DA, the BA, the EFWA, and the PSO

#### 3.2.1. Comparison of Optimization Accuracy

In this section, we compare the performance of the DA with the BA, the EFWA, and the PSO in terms of optimization accuracy.

Table 3 shows the optimization accuracy of the four algorithms on twelve benchmark functions, which are averaged over 51 independent runs. It can be seen that the proposed DA clearly outperforms among BA, EFWA, and PSO on most functions. In the function Six-Hump Camel-Back, the four algorithms almost achieve the same accuracy.

#### 3.2.2. Comparison of Convergence Speed

**Figure 2:**
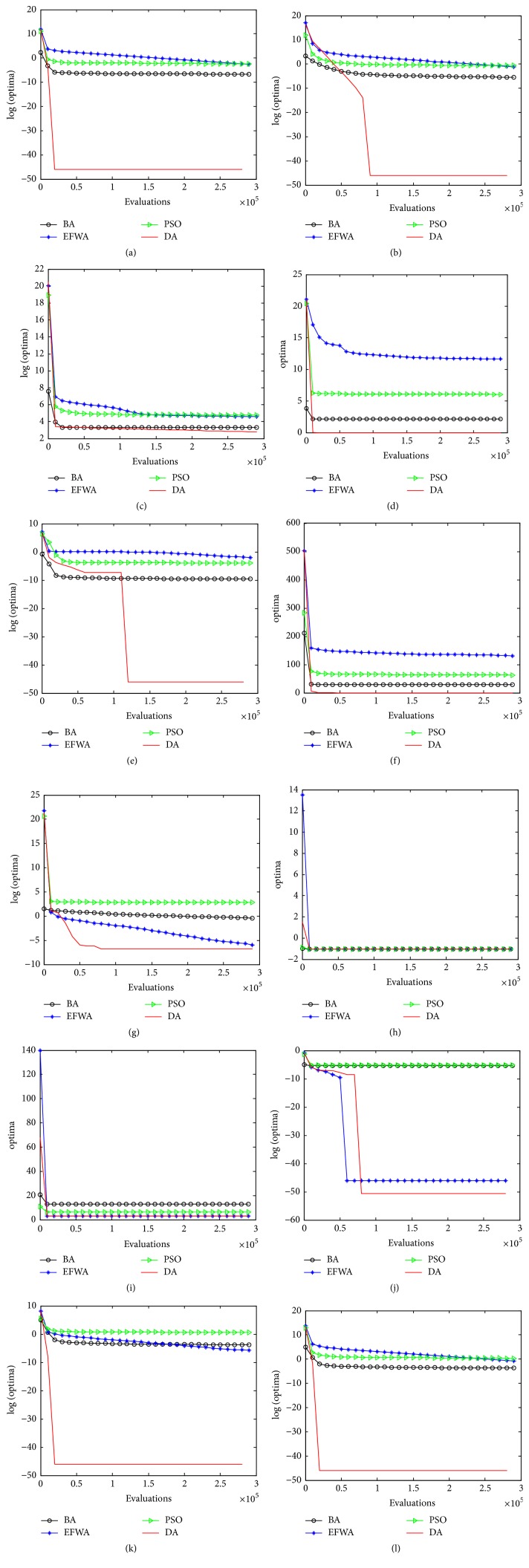
Convergence curves of the DA, the BA, the EFWA, and the PSO on twelve benchmark functions. (a) Sphere function; (b) Schwefel function; (c) Rosenbrock function; (d) Ackley function; (e) Griewank function; (f) Rastrigin function; (g) Penalized function; (h) Six-Hump Camel-Back function; (i) Goldstein-Price function; (j) Schaffer function; (k) Axis Parallel Hyper Ellipsoid function; (l) Rotated Hyper Ellipsoid function.

Besides optimization accuracy, convergence speed is quite essential to an optimizer. To validate the convergence speed of the DA, we conducted more thorough experiments.  Figure 2 shows the convergence curves of the DA, the BA, the EFWA, and the PSO on twelve benchmark functions averaged over 51 runs. From these results, in the function Six-Hump Camel-Back, the four algorithms have the same convergence speed, except for the fact that, in the other functions, we can arrive at a conclusion that the proposed DA has a much faster speed than the BA, the EFWA, and the PSO.

### 3.3. Discussion

As shown in the experiments, we can see that the proposed algorithm DA is a very promising algorithm. It is potentially more powerful than bat algorithm, particle swarm optimization, and enhanced fireworks algorithm. The primary reason lies in the following two aspects.

1. In the DA, the dandelion population is divided into two separate populations: core dandelion and assistant dandelion, and these two types of dandelion are applied in different ways to sow seeds. The two dandelion populations complement each other and coevolve to fully extend the search range, which increases the probability of finding the optimal location.
2. Two types of seeds are generated to avoid falling into local optimal and keep the diversity of seeds, and the selection strategy is a mechanism for keeping diversity. Therefore, the DA has the capability of avoiding premature convergence.

## 4. Optimization for Extreme Learning Machine with DA

### 4.1. Brief Introduction of Extreme Learning Machine

Extreme learning machine (ELM) is a neural network algorithm, which is proposed by Huang [21]. The biggest feature of ELM is faster and has better generalization performance than the traditional neural network learning algorithm (especially the single hidden layer feed-forward neural network).

For *N* arbitrary samples (*x*_*i*_, *y*_*i*_) and *x*_*i*_ = [*x*_*i*1_, *x*_*i*2_,…,*x*_*in*_]^T^ ∈ *R*^*n*^, *y*_*i*_ = [*y*_*i*1_, *y*_*i*2_,…,*y*_*in*_]^T^ ∈ *R*^*n*^. The output of the feed-forward neural network with *L* hidden layer nodes and the stimulus function *g*(*x*) can be expressed as

(10) $$\begin{array}{c} \overset{L}{\underset{i=1}{\sum }}\beta_{i}\cdot g\left(\;w_{i}\cdot x_{j}+a_{i}\;\right)=O_{j},\;j=1,\ldots ,N, \end{array}$$

where *w*_*i*_ is the single hidden layer input weight, *O*_*i*_ is the single hidden layer output weight, and *a*_*i*_ is the single hidden layer bias.

The purpose of neural network training is to minimize the error of the output value:

(11) $$\begin{array}{c} \overset{N}{\underset{j=1}{\sum }}\left\|\;O_{j}-y_{j}\;\right\|=0. \end{array}$$

From (11), we can see that there are *β*_*i*_, *w*_*i*_, *a*_*i*_ that make the following formula set up.

(12) $$\begin{array}{c} \overset{L}{\underset{i=1}{\sum }}\beta_{i}\cdot g\left(\;w_{i}\cdot x_{j}+a_{i}\;\right)=y_{j},\;j=1,\ldots ,N, \end{array}$$

expressed by the matrix as

(13) $$\begin{array}{c} H\beta =T. \end{array}$$

In the extreme learning machine, once the input weight *w*_*i*_ and the hidden layer bias *a*_*i*_ are randomly determined, the output matrix *H* of the hidden layer is uniquely determined. Then the training single hidden layer neural network is transformed into solving a linear equation *Hβ* = *T*, and the output weights can be determined, *β* = *H*^−1^*T*.

### 4.2. Optimization for Extreme Learning Machine with DA

In ELM, the single hidden layer input weights and bias are randomly generated based on the number of hidden layer nodes and neurons and then calculate the output weight matrix. Randomly generated input weights and bias are only a few of which are superior. And even part of the input weight and bias is 0, which leads to the result that the hidden layer node is invalid directly.

In order to solve the above problems of ELM, a new dandelion algorithm is proposed to optimize the ELM (DA-ELM). DA is a new evolutionary algorithm with strong advantages in accuracy and convergence performance. The DA chooses the best input weight and bias matrix by the iteration. The most suitable input weight and bias form a new matrix, and then the output weight matrix is calculated.

The specific steps of the DA-ELM algorithm are as follows.

**Step 1 .**

Set the initial parameters of the ELM, including the number of hidden layer nodes *L* and the stimulus function *g*(*x*).

**Step 2 .**

Initialize the parameters of the DA (refer to Table 1).

**Step 3 .**

Initialize the dandelion population and randomly generate the initial solution. The dimension of each solution is *L* × (*n* + 1) (*n* is the number of neurons). *L* × *n* dimension represents the input weight, and the remaining *L* dimension represents the hidden layer bias.

**Step 4 .**

Perform the dandelion algorithm to find the optimal solution, and the root mean square error (RMSE) calculated from the training sample is taken as the fitness function of the dandelion algorithm.

**Step 5 .**

Determine whether the DA has reached the maximum number of iterations, and if it is satisfied, go to Step 6; otherwise return to Step 4 to continue the algorithm.

**Step 6 .**

The optimal input weight and the hidden layer bias can be obtained by the returned optimal solution.

**Step 7 .**

Use the input weight value and the hidden layer bias to train the ELM.

### 4.3. Performance Evaluation

#### 4.3.1. Parameter Settings

In order to measure the relative performance of the DA-ELM, a comparison among the DA-ELM, ELM, PSO-ELM, BA-ELM, and EFWA-ELM is conducted on the biomedical datasets. The algorithms compared here are described as follows.

1. ELM: basic ELM with randomly generated hidden nodes and random neurons
2. PSO-ELM: using PSO to optimize for extreme learning machine
3. BA-ELM: using BA to optimize for extreme learning machine
4. EFWA-ELM: using EFWA to optimize for extreme learning machine

In this simulation, the performance of DA-ELM is evaluated on 4 real biomedical datasets classification problems from the UCI database, namely, the EEG Eye State dataset (EEG), the Blood Transfusion Service Center dataset (Blood), the Statlog (Heart) dataset (Statlog), and the SPECT Heart dataset (SPECT). The following lists a detailed description of these 4 biomedical datasets.

1. EEG: the dataset consists of 14 EEG values and a value indicating the eye state.
2. Blood: the dataset is taken from the Blood Transfusion Service Center in Hsin-Chu City in Taiwan.
3. Statlog: this dataset concerns the presence of heart disease in the patient by using 13 attributes.
4. SPECT: data on cardiac Single Proton Emission Computed Tomography (SPECT) images, each patient classified into two categories: normal and abnormal.

The specification of these 4 datasets is shown in Table 4. All the attributes (inputs) have been normalized to the range of [−1,1] in our simulations and, for each trial of simulations, the training set and testing set are randomly generated from the whole dataset with the partition number shown in Table 4.

The parameters of the BA, the EFWA, the PSO, and the DA are setting as Table 1, and the algorithms all have 1000 function evaluations. In our experiments, we set the the number of hidden layer nodes *L* = 20 and set the stimulus function as “sigmoid,” and the experimental results are the average of the algorithm running 10 times.

All these simulations are conducted in Matlab R2014a software on a PC with a 3.2 GHz CPU (Intel Core i5-3470), and 4 GB RAM, and Windows 7 (64 bit).

#### 4.3.2. Optimization of ELM by Dandelion Algorithm for Biomedical Classification

In this section, we propose a new method to optimize the extreme learning machine by using the DA. The DA is used to optimize the input weight and the hidden layer bias of ELM. Combining the advantages of DA and ELM, the algorithm of DA-ELM is proposed.

The averaging classification results of multiple trials for all these four datasets are shown in Table 5. The one with the best testing rate or the best deviation is shown in boldface. We can easily find that the DA-ELM has higher classification accuracy and better stability among five algorithms in the biomedical classification problems.

#### 4.3.3. Comparison between DA-ELM and Fusion Classifiers for Biomedical Classification

In order to further improve the accuracy and stability of the classification, we combine five classifiers to form a fusion classifier. There are some fusion methods available, such as majority voting method [22], maximum method [22], minimum method [22], median method [22], a new method for fusing scores corresponding to different detectors [23], and fusion of nonindependent detectors [24]. Here we select some simple and effective fusion methods to form fusion classifiers. The classifiers compared here are described as follows.

1. Max-ELM: fusion of DA-ELM, PSO-ELM, ELM, EFWA-ELM, and BA-ELM to form a fusion classifier and the fusion classifier to make decisions with maximum method
2. Min-ELM: fusion of DA-ELM, PSO-ELM, ELM, EFWA-ELM, and BA-ELM to form a fusion classifier and the fusion classifier to make decisions with minimum method
3. Med-ELM: fusion of DA-ELM, PSO-ELM, ELM, EFWA-ELM, and BA-ELM to form a fusion classifier and the fusion classifier to make decisions with median method
4. MV-ELM: fusion of DA-ELM, PSO-ELM, ELM, EFWA-ELM, and BA-ELM to form a fusion classifier and the fusion classifier to make decisions with majority voting method

The averaging classification results for DA-ELM and the four fusion classifiers are shown in Table 6. The one with the best testing rate or the best deviation is shown in boldface. We can find that the Max-ELM (fusion classifier) has achieved the higher accuracy and the smallest deviation in these datasets, and the Max-ELM has better stability than other fusion methods and DA-ELM.

## 5. Conclusions

The major contribution of this article is to propose a new dandelion algorithm (DA) for function optimization and optimize the extreme learning machine for biomedical classification problems. From the test results, it is found that the DA can usually find solutions correctly and it clearly outperforms the BA, the EFWA, and the PSO on twelve benchmark functions in terms of both optimization accuracy and convergence speed. Moreover, we use DA to handle the ELM optimization; the results of the ELM optimization also showed that the DA has high performance in unknown, challenging search spaces. At last, we combine five classifiers to form different fusion classifiers with different fusion methods, and the results show that the fusion classifier (Max-ELM) not only has a relatively high accuracy but also has better stability.

For future work, we will seek a deep theoretical analysis on the DA and try to apply the DA to more practical engineering applications. However, the DA is proposed by this article might not be thorough, and we hope that more researchers can participate in the promotion and test sincerely. Moreover, we will combine other neural networks with DA-ELM to achieve higher classification accuracy and better stability.

## Figures and Tables

**Algorithm 1:**
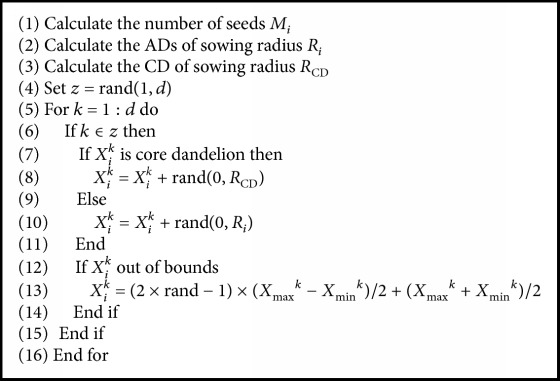
Generating normal seeds.

**Algorithm 2:**
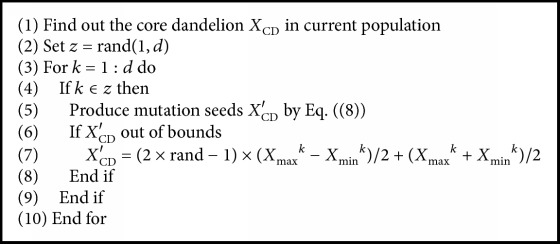
Generating mutation sparks.

**Algorithm 3:**
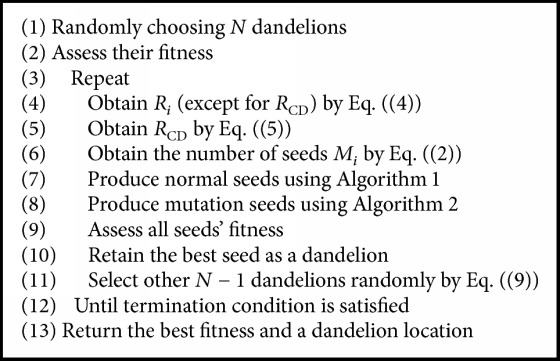
Framework of DA.

**Table 1:** Parameter settings.

Algorithm	Parameters
BA	*n* = 20, *A* = 1, *r* = 1, *α* = *γ* = 0.9
EFWA	*n* = 50, *m* = 50, *a* = 0.8, *b* = 0.04, *A*_max_ = 40
PSO	*n* = 20, *c*1 = 2, *c*2 = 2, *w* = 0.7298
DA	*n* = 2, *n*_*m*_ = 2, max = 100, min = 10, *r* = 0.95, *e* = 1.05

**Table 2:** Twelve benchmark functions utilized in our experiments.

Function	Range	Optimal value	Dimension
Sphere	[−100,100]	0	30
Schwefel	[−100,100]	0	30
Rosenbrock	[−30,30]	0	30
Ackley	[−32,32]	0	30
Griewank	[−600,600]	0	30
Rastrigin	[−5.12,5.12]	0	30
Penalized	[−50,50]	0	30
Six-Hump Camel-Back	[−5,5]	−1.032	2
Goldstein-Price	[−2,2]	3	2
Schaffer	[−100,100]	0	2
Axis Parallel Hyper Ellipsoid	[−5.12,5.12]	0	30
Rotated Hyper Ellipsoid	[−65.536,65.536]	0	30

**Table 3:** Mean value and standard deviation achieved by DA, BA, EFWA, and PSO (accurate to 10^−6^).

Function	BA mean (Std)	EFWA mean (Std)	PSO mean (Std)	DA mean (Std)
Sphere	0.001277 (0.000144)	0.079038 (0.010276)	0.09323 (0.053308)	0.000000 (0.000000)
Schwefel	0.00417 (0.000856)	0.310208 (0.082243)	0.551331 (0.382977)	0.000000 (0.000000)
Rosenbrock	26.94766 (1.396553)	97.43135 (86.30464)	117.0419 (100.6725)	15.88892 (0.262501)
Ackley	2.175684 (0.386022)	11.67335 (9.79794)	6.062462 (1.350912)	0.000000 (0.000000)
Griewank	0.000069 (0.000009)	0.139219 (0.027736)	0.020483 (0.019879)	0.000000 (0.000000)
Rastrigin	29.47549 (7.795089)	130.8502 (22.96112)	63.04136 (15.74571)	0.000000 (0.000000)
Penalized	0.673172 (0.804593)	0.002687 (0.001646)	17.48197 (11.78603)	0.001939 (0.00423)
Six-Hump Camel-Back	−1.03163 (0.000000)	−1.03163 (0.000000)	−1.03163 (0.000000)	−1.03163 (0.000000)
Goldstein-Price	13.05883 (18.67556)	3.000000 (0.000000)	6.176471 (15.87918)	3.000000 (0.000000)
Schaffer	0.004731 (0.006673)	0.000000 (0.000000)	0.005302 (0.00666)	0.000000 (0.000000)
Axis Parallel Hyper Ellipsoid	0.023513 (0.004702)	0.00306 (0.000568)	1.743886 (1.521884)	0.000000 (0.000000)
Rotated Hyper Ellipsoid	0.024743 (0.005611)	0.490085 (0.073349)	1.30426 (2.23374)	0.000000 (0.000000)

**Table 4:** Biomedical datasets.

Datasets	Data		Type	Attributes	Classes
	Training	Testing			
EEG	7490	7490	Classification	14	2
Blood	374	374	Classification	4	2
Statlog	135	135	Classification	13	2
SPECT	133	134	Classification	44	2

**Table 5:** Results comparisons for biomedical classification.

Datasets	Algorithms	Training		Testing	
		Rate (%)	Dev	Rate (%)	Dev
EEG	DA-ELM	69.78	0.0052	**70.22**	0.0062
	ELM	63.51	0.0167	63.74	0.0139
	PSO-ELM	69.64	0.0069	70.06	0.0064
	BA-ELM	68.19	0.0098	68.79	0.0078
	EFWA-ELM	68.76	0.0068	68.82	0.0072

Blood	DA-ELM	79.81	0.0140	**81.68**	0.0133
	ELM	80.64	0.0162	78.64	0.0133
	PSO-ELM	80.83	0.0131	80.40	0.0141
	BA-ELM	79.97	0.0174	80.16	0.0159
	EFWA-ELM	80.70	0.0175	79.39	0.0154

Statlog	DA-ELM	86.22	0.0216	**88.15**	0.0175
	ELM	87.11	0.0286	80.52	0.0268
	PSO-ELM	86.74	0.0285	88.07	0.0228
	BA-ELM	84.96	0.0257	87.26	0.0167
	EFWA-ELM	87.11	0.0264	86.37	0.0177

SPECT	DA-ELM	81.35	0.0255	**85.22**	0.0243
	ELM	80.68	0.0313	80.75	0.0245
	PSO-ELM	81.95	0.0271	85.00	0.0370
	BA-ELM	80.00	0.0286	84.25	0.0250
	EFWA-ELM	80.98	0.0292	84.33	0.0354

**Table 6:** Results comparisons between DA-ELM and fusion classifier for biomedical classification.

Datasets	Algorithms	Training		Testing	
		Rate (%)	Dev	Rate (%)	Dev
EEG	DA-ELM	69.78	0.0052	70.22	0.0062
	Max-ELM	70.13	0.005	**70.58**	**0.0053**
	Min-ELM	68.97	0.0087	69.72	0.0062
	Med-ELM	69.93	0.0063	70.42	0.0059
	MV-ELM	69.14	0.0091	69.13	0.0056

Blood	DA-ELM	79.81	0.0140	81.68	0.0133
	Max-ELM	81.63	0.0138	**81.96**	**0.0125**
	Min-ELM	80.56	0.0139	81.73	0.0142
	Med-ELM	80.79	0.0142	81.81	0.0131
	MV-ELM	79.06	0.0168	81.04	0.0125

Statlog	DA-ELM	86.22	0.0216	88.15	0.0175
	Max-ELM	87.16	0.0208	**89.95**	**0.0137**
	Min-ELM	86.56	0.0213	88.62	0.0142
	Med-ELM	86.75	0.0218	88.16	0.0151
	MV-ELM	88.74	0.0209	87.56	0.0143

SPECT	DA-ELM	81.35	0.0255	85.22	0.0243
	Max-ELM	81.56	0.0209	**86.83**	**0.0226**
	Min-ELM	81.73	0.0226	86.52	0.0235
	Med-ELM	81.25	0.0237	85.69	0.0239
	MV-ELM	80.68	0.0213	84.48	0.0232

## Appendix

The expression of the Sphere function is

(A.1) $$\begin{array}{c} f\left(\;x\;\right)=\overset{D}{\underset{i=0}{\sum }}{x_{i}}^{2}. \end{array}$$

The expression of the Schwefel function is

(A.2) $$\begin{array}{c} f\left(\;x\;\right)={\sum_{i=1}^{D}\left(\sum_{j=1}^{i}x_{j}\right)}^{2}. \end{array}$$

The expression of the Rosenbrock function is

(A.3) $$\begin{array}{c} f\left(\;x\;\right)=\overset{D-1}{\underset{i=1}{\sum }}\left(\;100{\left(\;x_{i+1}-{x_{i}}^{2}\;\right)}^{2}+{\left(\;x_{i}-1\;\right)}^{2}\;\right). \end{array}$$

The expression of the Ackley function is

(A.4) fx=−20exp⁡−0.21D∑i=1Dxi2−exp⁡1D∑i=1Dcos⁡2πxi+20+e.

The expression of the Griewank function is

(A.5) $$\begin{array}{c} f\left(\;x\;\right)=1+\overset{D}{\underset{i=1}{\sum }}\frac{{x_{i}}^{2}}{4000}+\overset{D}{\underset{i=1}{\prod }}\cos\left(\;\frac{x_{i}}{\sqrt{i}}\;\right). \end{array}$$

The expression of the Rastrigin function is

(A.6) $$\begin{array}{c} f\left(\;x\;\right)=\overset{D}{\underset{i=1}{\sum }}\left(\;{x_{i}}^{2}-10\cos\left(\;2\pi x_{i}\;\right)+10\;\right). \end{array}$$

The expression of the Penalized function is

(A.7) fx=0.1sin2⁡3πx1+∑i=1D−1xi−121+sin2⁡3πxi+1+xD−121+sin2⁡2πxD+∑i=1Duxi,5,100,4.

The expression of the Six-Hump Camel-Back function is

(A.8) $$\begin{array}{c} f\left(\;x\;\right)=4{x_{1}}^{2}-2.1{x_{1}}^{4}+\frac{{x_{1}}^{6}}{3}+x_{1}x_{2}-4{x_{2}}^{2}+4{x_{2}}^{4}. \end{array}$$

The expression of the Goldstein-Price function is

(A.9) fx=1+x1+x2+1219−14x1+3x12−14x2+6x1x2+3x22·30+2x1−3x22·18−32x1+12x12+48x2−36x1x2+27x22.

The expression of the Schaffer function is

(A.10) $$\begin{array}{c} f\left(\;x\;\right)=\frac{\sin^{2}\sqrt{{x_{1}}^{2}+{x_{2}}^{2}}-0.5}{{\left[1+0.001\left({x_{1}}^{2}+{x_{2}}^{2}\right)\right]}^{2}}. \end{array}$$

The expression of the Axis Parallel Hyper Ellipsoid function is

(A.11) $$\begin{array}{c} f\left(\;x\;\right)=\overset{D}{\underset{i=0}{\sum }}i{x_{i}}^{2}. \end{array}$$

The expression of the Rotated Hyper Ellipsoid function is

(A.12) $$\begin{array}{c} f\left(\;x\;\right)={\sum_{i=1}^{D}\left(\sum_{j=1}^{i}{x_{j}}^{2}\right)}^{2}. \end{array}$$
